# Epigenome-Wide Histone Acetylation Changes in Peripheral Blood Mononuclear Cells in Patients with Type 2 Diabetes and Atherosclerotic Disease

**DOI:** 10.3390/biomedicines9121908

**Published:** 2021-12-14

**Authors:** Pradeep Bompada, Isabel Goncalves, Chuanyan Wu, Rui Gao, Jiangming Sun, Bilal Ahmad Mir, Cheng Luan, Erik Renström, Leif Groop, Jianping Weng, Ola Hansson, Andreas Edsfeldt, Yang De Marinis

**Affiliations:** 1 Department of Clinical Sciences, Lund University, 20502 Malmö, Sweden; bompada.pradeep@med.lu.se (P.B.); chuanyan.wu@med.lu.se (C.W.); bilal.mir@med.lu.se (B.A.M.); cheng.luan@med.lu.se (C.L.); erik.renstrom@rektor.lu.se (E.R.); leif.groop@med.lu.se (L.G.); ola.hansson@med.lu.se (O.H.); 2 Cardiovascular Research-Translational Studies, Institution of Clinical Science Malmö, Lund University, 20502 Malmö, Sweden; migvdias@gmail.com (I.G.); jiangming.sun@med.lu.se (J.S.); andreas.edsfeldt@med.lu.se (A.E.); 3 Department of Cardiology, Skåne University Hospital, 20502 Malmö, Sweden; 4 School of Control Science and Engineering, Shandong University, Jinan 250061, China; 5 School of Intelligent Engineering, Shandong Management University, Jinan 250100, China; 6 Finnish Institute for Molecular Medicine, University of Helsinki, 00290 Helsinki, Finland; 7 Clinical Research Hospital, Chinese Academy of Sciences, Hefei 230001, China; wengjp@ustc.edu.cn; 8 Department of Endocrinology and Metabolism, Division of Life Sciences of Medicine, University of Science and Technology of China, Hefei 230001, China; 9 Institute for Molecular Medicine Finland (FIMM), Helsinki University, 00290 Helsinki, Finland; 10 Wallenberg Center for Molecular Medicine, Lund University, 20502 Malmö, Sweden

**Keywords:** type 1 diabetes, type 2 diabetes, histone modification, ChIP-seq, H3K9ac, *TCF7L2*, *HLA*

## Abstract

There is emerging evidence of an association between epigenetic modifications, glycemic control and atherosclerosis risk. In this study, we mapped genome-wide epigenetic changes in patients with type 2 diabetes (T2D) and advanced atherosclerotic disease. We performed chromatin immunoprecipitation sequencing (ChIP-seq) using a histone 3 lysine 9 acetylation (H3K9ac) mark in peripheral blood mononuclear cells from patients with atherosclerosis with T2D (*n* = 8) or without T2D (ND, *n* = 10). We mapped epigenome changes and identified 23,394 and 13,133 peaks in ND and T2D individuals, respectively. Out of all the peaks, 753 domains near the transcription start site (TSS) were unique to T2D. We found that T2D in atherosclerosis leads to an H3K9ac increase in 118, and loss in 63 genomic regions. Furthermore, we discovered an association between the genomic locations of significant H3K9ac changes with genetic variants identified in previous T2D GWAS. The transcription factor 7-like 2 (*TCF7L2*) rs7903146, together with several human leukocyte antigen (*HLA*) variants, were among the domains with the most dramatic changes of H3K9ac enrichments. Pathway analysis revealed multiple activated pathways involved in immunity, including type 1 diabetes. Our results present novel evidence on the interaction between genetics and epigenetics, as well as epigenetic changes related to immunity in patients with T2D and advanced atherosclerotic disease.

## 1. Introduction

Atherosclerosis accounts for more than 80% of deaths among patients with diabetes. Strong evidence from large treatment studies, such as the United Kingdom Prospective Diabetes Study (UKPDS) and Diabetes Control and Complications Trial (DCCT), supports an association between glycemic control and cardiovascular disease (CVD) risk [1,2]. Animal and human studies have provided further evidence that prolonged exposure to hyperglycemia induces alterations in vascular tissue that potentially accelerate the atherosclerotic process [3,4]. The atherogenic role of glucose involves protein and lipid glycosylation relevant to the atherosclerotic process, oxidative stress and protein kinase C (PKC) activation [5]. There is also emerging evidence of associations between epigenetic modifications and atherosclerosis risk.

Epigenetic mechanisms involve interactions between environmental factors (e.g., hyperglycemia) and gene expression via altering DNA methylation, non-coding RNAs, and histone modifications. These processes may persist for a lifetime and even be heritable. Histone acetylation contributes to the regulation of gene expression through its effect on conformational changes in chromatin. Histone 3 lysine 9 (H3K9ac) is a frequently acetylated site in active gene transcription under hyperglycemia. Previous investigations, including our own studies, have shown that H3K9ac is an important modification for transcription activity in glucotoxicity [6,7,8].

Histone acetylation is a dynamic process regulated by histone acetyl-transferases (HATs) and histone deacetylases (HDACs), which add and remove acetyl groups, respectively. HATs transfer acetyl groups generated from acetyl-coenzyme A (acetyl-CoA) to lysine residues on histone tails. Glucose is one of the major sources for the production of acetyl-CoA via the tricarboxylic acid cycle [9,10,11,12]; therefore, the availability of acetyl-CoA and thereby acetyl groups, also contributes to the levels of histone acetylation. Previous studies have shown that global histone acetylation levels in T2D patients are higher when compared to healthy controls, and are often associated with increases in gene expression [9,13,14]. Glucose-induced H3K9ac is found to be involved in the upregulation of glucotoxicity-related genes such as *NF-kB* and *TXNIP* in blood monocytes, pancreatic islet cells and kidney cells [7,15,16]. Therefore, we hypothesized that epigenetic regulation by H3K9ac could also be involved in T2D patients suffering from atherosclerosis.

In this study, we compared the genome-wide profiles of H3K9ac in peripheral blood mononuclear cells (PBMCs) of atherosclerotic patients with or without T2D, to elucidate key epigenetic mechanisms underlying T2D in atherosclerosis.

## 2. Materials and Methods

### 2.1. Carotid Plaque Imaging Project (CPIP) Cohort

The CPIP cohort is designed to study atherosclerosis and inflammatory or immune markers to identify mechanisms in atherosclerotic plaques that lead to the development of myocardial infarctions or strokes. The CPIP is an ongoing study since November 2005 that recruits patients who undergo carotid endarterectomy at Lund University Hospital, Malmö, Sweden. Blood samples were drawn from patients a day before the surgery. The criteria for surgery are (1) stroke, transient ischemic attack or amaurosis fugax and a stenosis degree (assessed by ultrasound) of >70%, or (2) no symptoms and a stenosis degree of >80%. For the current study, we studied blood PBMCs from 18 patients with (*n* = 8) or without (*n* = 10) T2D. The patients’ characteristics are summarized in Table 1. Serum C-peptide levels were measured using ELISA (10-1136-01, Mercodia, Uppsala, Sweden).

All patients gave informed consent and the study has been approved by the Lund/Malmö ethical committee (Approval Number and date: 472/2005-8 September 2005, 2014/904-14 December 2014). All experiments were performed in accordance with relevant guidelines and regulations.

### 2.2. PBMCs Isolation

Five milliliters of an EDTA-blood sample were used to isolate PBMC using Ficoll Paque Plus (GE17-1440-02) density gradient centrifugation. The total volume of blood was layered on 2.5 mL Ficoll Paque Plus and centrifuged at 1350× *g* for 10 min without braking to form gradients. The upper most layer was plasma, followed by the PBMC cell layer, the ficoll layer and granulocytes. Carefully, the PBMC layer was collected and washed by adding 0.9% NaCl, centrifuged at 600× *g* for 10 min. The washing step was repeated by adding NaCl, and followed by centrifugation at 300× *g* for 10 min. Finally, cells were suspended in 500 µL autologous plasma and cells were counted using a Burkes chamber. Cells were stored in freezing media containing 20% DMSO in RPMI1640 in liquid N_2_.

### 2.3. Chromatin Immunoprecipitation (ChIP)

ChIP was performed as previously described [7,17]. PBMCs were cross-linked by formaldehyde (final concentration 1%) and sonicated by a Bioruptor sonicator (Diagenode, Denville, NJ, USA) for 25 cycles of 30 s with a 30 s interval (medium intensity) period between cycles. Genomic DNA fragment lengths of 200–1000 bp were achieved after sonication. The lysates were then centrifuged, and the supernatants were collected. A 10% volume of each sample was set aside as the input control. The sonicated chromatin was incubated overnight at 4 °C with a 1 µg antibody binding to histone H3 lysine 9-acetylated (H3K9ac, ab4441, Abcam, Cambridge, United Kingdom). DNA–protein complexes were captured with 15 µL of 50% protein G beads, followed by reverse cross-linking and protease K digestion. The DNA fragments were purified using a MinElute PCR Purification Kit (Qiagen, Hilden, Germany).

### 2.4. Library Preparation

The purified DNA was then processed including end repair: A-tailing and barcode adapter ligation (Nextflex-HT barcodes 228-514174, Nextflex, San Jose, CA, USA). DNA libraries were then sequenced on Illumina HiSeq 2000.

### 2.5. ChIP-Seq Analysis

ChIP seq was performed with 40 M effective reads. The ingroup standard deviation among replicates was set at 10% of the average read density. ChIP-seq tags generated with the Illumina HiSeq platform were de-multiplexed with the bcl2fastq utility and aligned to the human reference genome (assembly NCBI37/hg19) using BWA v0.7.10, allowing up to three mismatches per sequencing tag (default parameters). Peaks were detected using MACS2 v2.1.1 (tag size = 100 bp; false discovery rate (FDR) <1 × 10^−3^) from pooled H3K9ac tags of patients with each individual’s input tags as control. Within each pooled sample, peaks whose termini were within 150 bp were merged into one peak. The MAnorm method was then used to compare H3K9ac enrichment across the two study groups. MAnorm took the coordinate of all peaks and aligned reads in both group samples as input. The (M, A) value of each common peak was then calculated and plotted, where M = log_2_ (Read density in sample 1/Read density in sample 2) and A = 0.5 × log_2_ (Read density in sample 1 × Read density in sample 2). A robust regression was subsequently applied to the (M, A) values of all common peaks and a linear model was derived. Finally, the linear model was extrapolated to all peaks for normalization. For each peak, a *p*-Value was calculated to examine the statistical significance (<0.001) of read intensity difference between the samples from the two groups. The *p*-value calculation was based on a Bayesian model developed by Audic and Claverie [18]. The value of M describes the log_2_ fold change of the read density at a peak region between two samples, and was used for downstream analysis (i.e., ND vs. T2D). Scatter plots, histograms, and box plots of ChIP-seq data were visualized using R software. Representative peaks at each gene that had significantly increased or decreased enrichment were generated by IGV software (GNU LGPL open-source, http://www.broadinstitute.org/igv (accessed on 28 September 2021), Broad Institute of MIT and Harvard, Cambridge, MA, USA).

### 2.6. Statistical Analysis

Statistical analysis of ChIP-seq data was performed by a Welch’s test. A Mann–Whitney test was used for clinical comparison of the patients from the ND and T2D groups. *p* < 0.05 were considered to be statistically significant.

### 2.7. Data and Resource Availability

Data presented in this manuscript are available upon reasonable request to the corresponding authors, with the exception of sensitive data according to current GDPR regulations.

## 3. Results

### 3.1. Genome-Wide Distribution of H3K9ac in Atherosclerosis Patients with T2D

**Figure 1:**
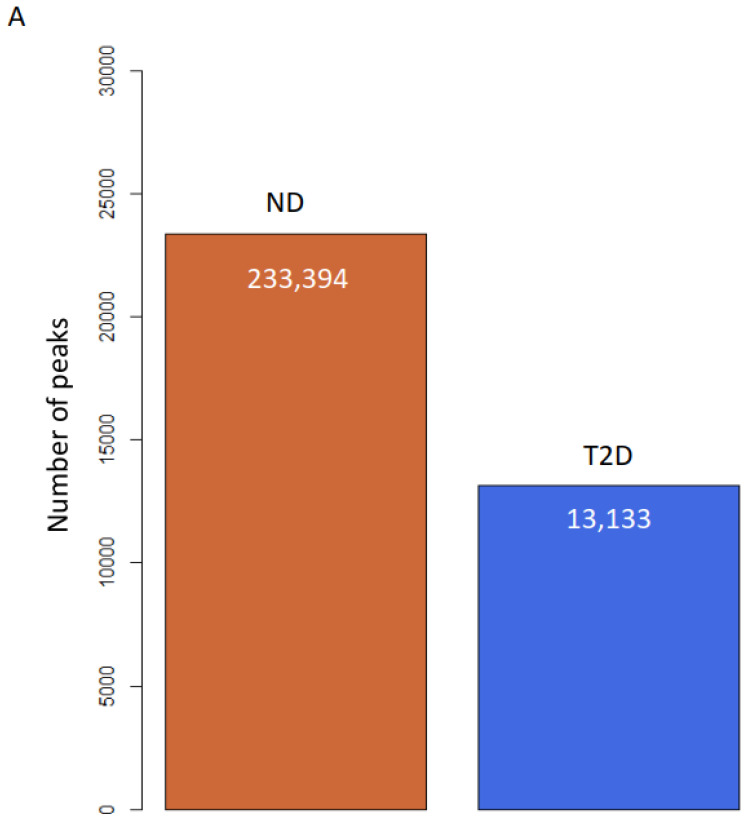

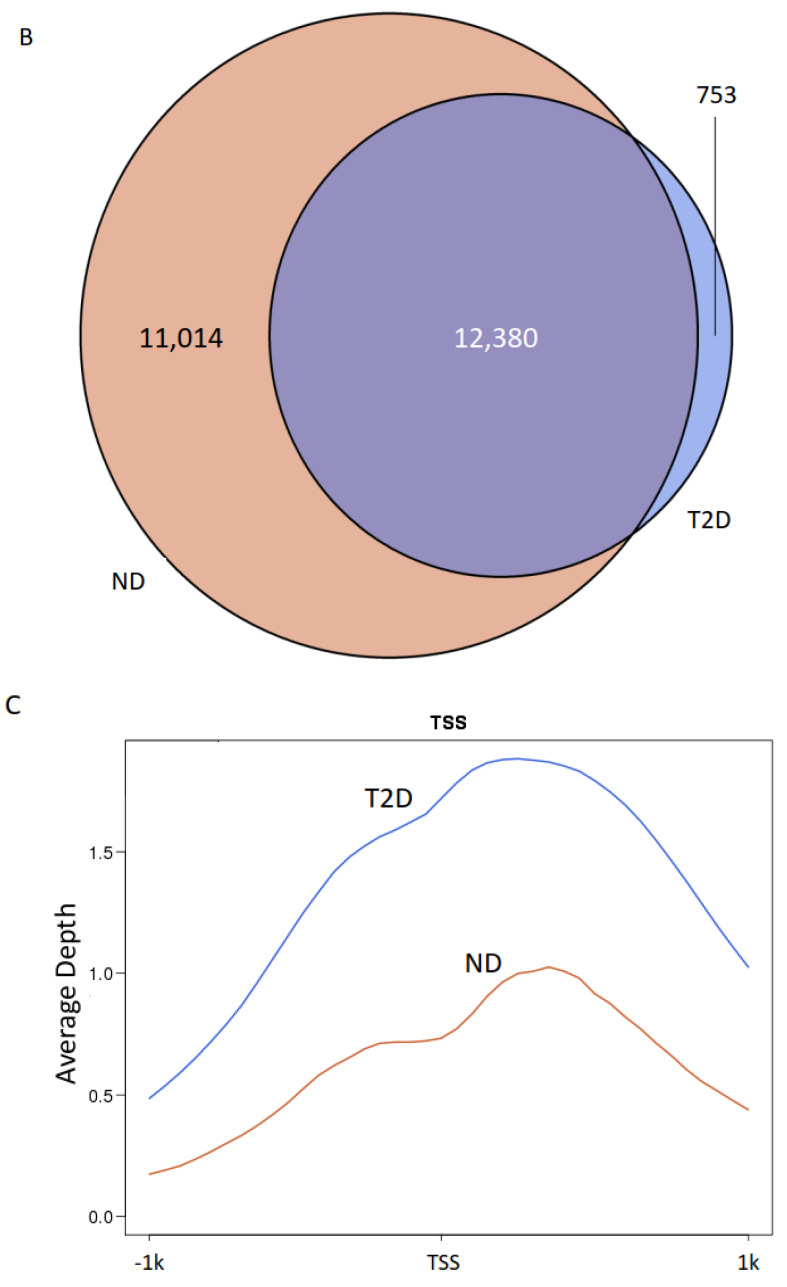

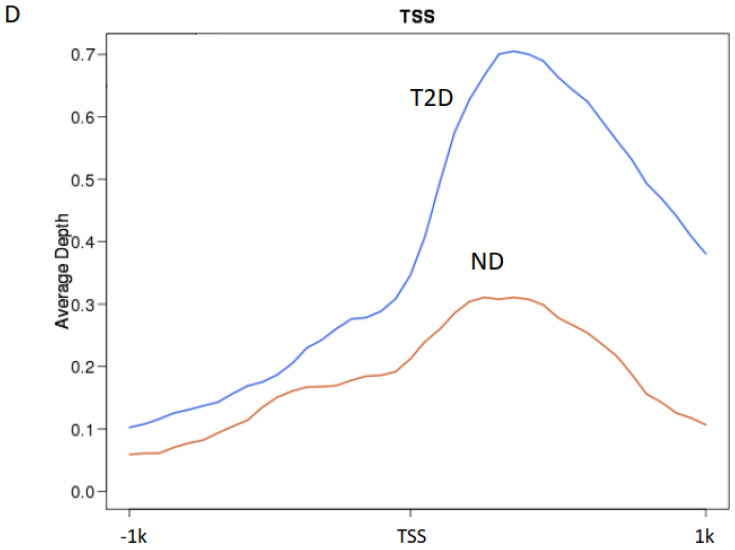
H3K9ac is redistributed in atherosclerosis patients with T2D. (**A**) Bar plot of total number of H3K9ac peaks. (**B**) Venn diagram of peak overlapping between ND (red) and T2D (blue). (**C**,**D**) H3K9ac enrichment at (**C**) TSSs (±1 kb) of constitutive peaks; (**D**) TSS (± 1 kb) where no peak was detected.

To study the role of H3K9ac in atherosclerosis patients with T2D, we profiled the genome-wide enrichment of H3K9ac by ChIP-seq in the PBMCs collected from patients with T2D (*n* = 8) or without T2D (ND, *n* = 10), all with advanced atherosclerotic disease. The H3K9ac peaks in each group were detected by the MACS2 peak calling method (FDR < 1 × 10^−3^). Differential peak enrichment was evaluated by assessing the enrichment of the corresponding region in individual samples. ChIP-seq peak calling detected 23,394 peaks in subjects without T2D and 13,133 peaks in subjects with T2D (Figure 1A). The difference in detected peaks indicates an overall decrease in the total number of H3K9ac peaks in T2D, which suggests a downregulation of H3K9ac in the T2D condition. There are 12,380 peaks common to both ND and T2D individuals (Figure 1B). When the constitutive peaks are compared to the remaining peaks in the T2D group, around 6% (753 peaks) of the peaks were redistributed in T2D, which may suggest different chromatin states in T2D (Figure 1B). We then compared the enrichment of the constitutive H3K9ac peaks around the transcriptional start sites (TSSs, ±1 kb), with that of the TSSs with no H3K9ac peaks. The levels of H3K9ac acetylation at the TSSs of constitutive peaks and no peaks-calling were both higher in T2D (Figure 1C,D). The TSSs of constitutive peaks showed a bimodal distribution in both +1kb and −1kb around the TSS (Figure 1C). In the TSSs where no H3K9ac peaks were called, distribution of H3K9ac acetylation was mostly enriched in +1kb of the TSSs (Figure 1D).

### 3.2. The Dynamics of the H3K9ac Changes in T2D

Quantification and comparison of the number of peaks that were gained or lost in ND and T2D showed 753 peaks gained in T2D, whereas no loss in peaks was detected (Figure 1B). This analysis suggests that H3K9ac tends to gain in T2D. To better understand the dynamics of the H3K9ac changes in T2D, we next performed quantitative measurements of H3K9ac enrichment.

**Figure 2:**
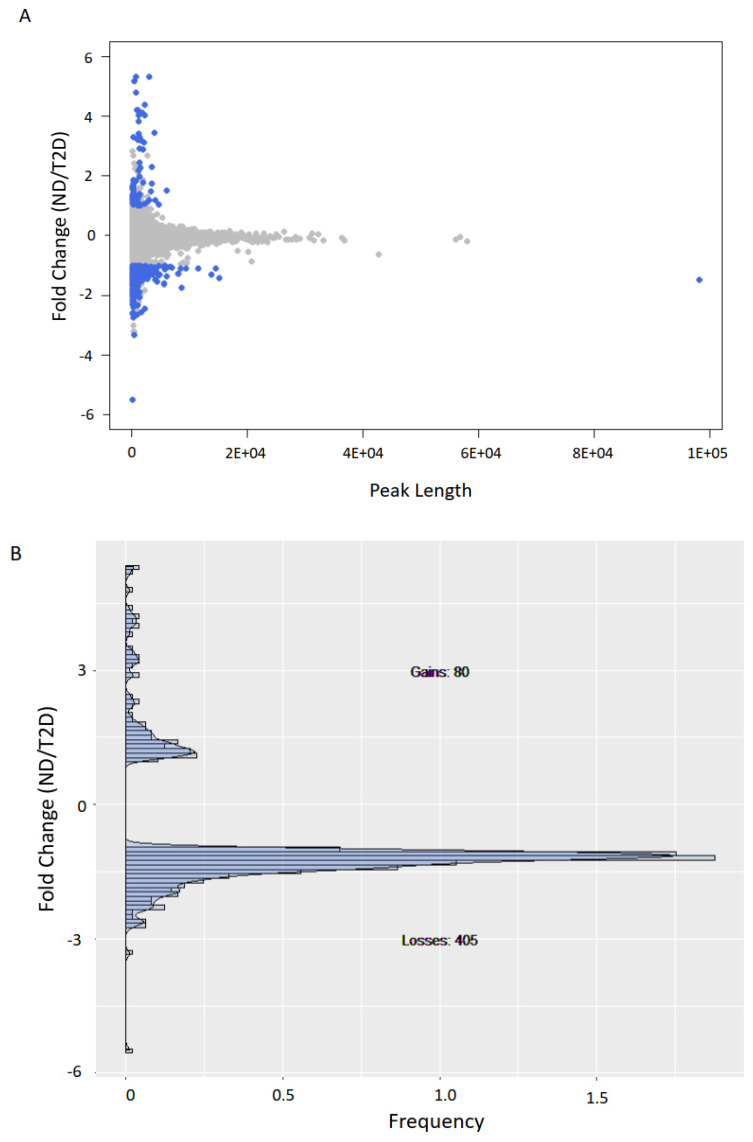
H3K9ac is predominantly lost in T2D. (**A**) Scatter plot of H3K9ac fold-change vs. peak size average (measured as area under the curve or AUC) for ND vs. T2D. (**B**) Histogram of H3K9ac fold-change vs. frequency for peaks with significant (*p* < 0.05, Welch’s t test, two-sided) H3K9ac changes (blue dots in (**A**)).

Individual heterogeneity could potentially impact variable peaks; therefore, to ensure that the observed trends were statistically significant, for each peak detected in ND or T2D individuals, we quantified the corresponding area under the curve in each patient and compared it with both ND and T2D groups. When comparing ND with T2D, we detected 80 peaks with a significant increase and 405 peaks with a significant decrease in H3K9ac with T2D (*p* < 0.05, Welch’s t test; Figure 2A,B).

### 3.3. H3K9ac-Enriched Genomic Regions in T2D Coincide with Genetic Loci Associated with T2D and Type 1 Diabetes (T1D)

**Figure 3:**
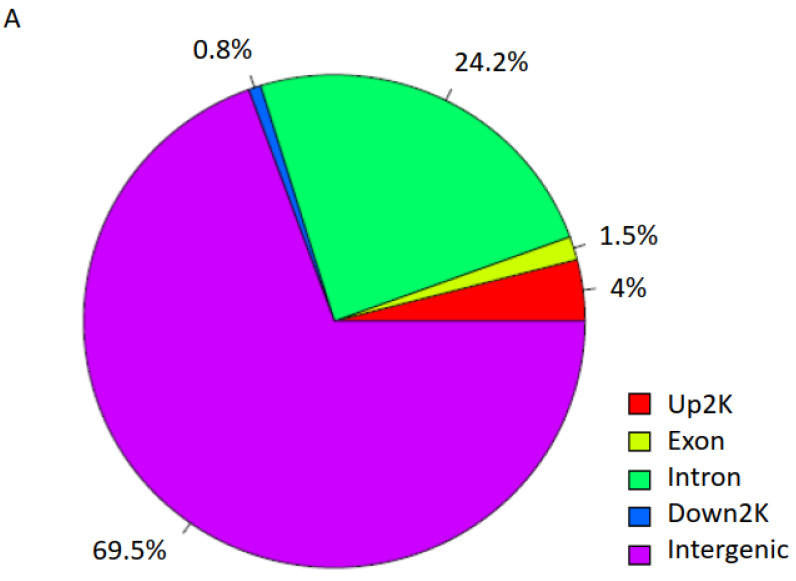

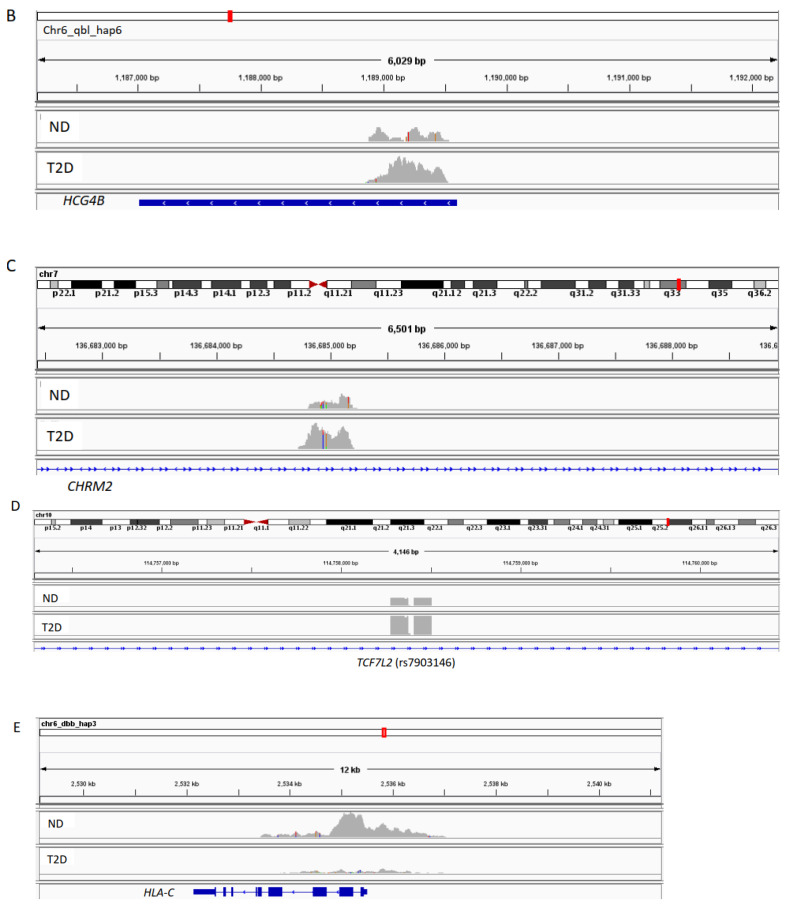

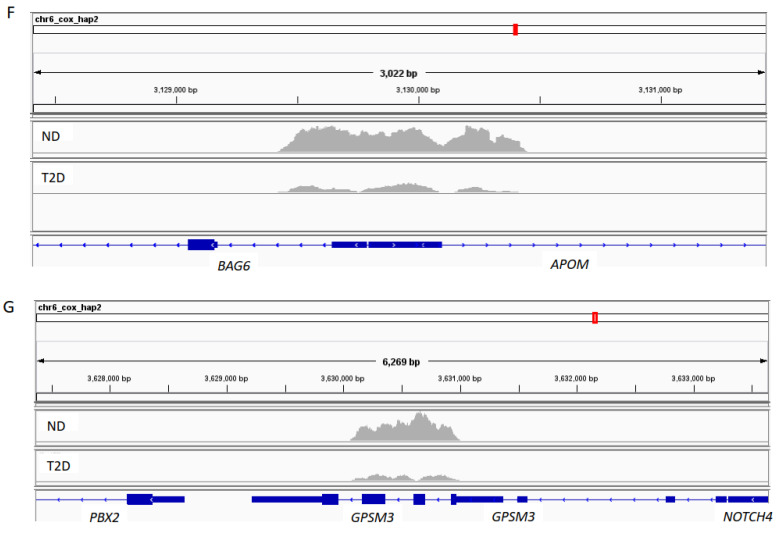
H3K9ac-enriched genomic regions in T2D. (**A**) Genomic region distribution of H3K9ac enrichment in T2D. (**B**–**G**) Representative UCSC Genome browser track views of H3K9ac changes in respective genes.

We then mapped and profiled T2D H3K9ac enrichment in annotated genomic regions. A comparison of ND and T2D H3K9ac enrichment revealed 181 genomic regions unique to T2D (top 10 loci displayed in Table 2 and Table 3; full gene list presented in Table S1). About 69.5% of the differentially enriched regions fall into intergenic regions; 24.2% in introns; 4% upstream and 0.8% downstream of the TSS; and 1.5% in exons (Figure 3A). Among these genes, 118 displayed increased (Tables S1 and S2) and 63 showed decreased H3K9ac enrichment in T2D (Table 3 and Table S1). Representative UCSC Genome browser track views of H3K9ac changes are presented in Figure 3B–G (B–D: increased H3K9ac; and E–G: decreased H3K9ac in T2D). The transcription factor 7-like 2 *(TCF7L2)* polymorphism at rs7903146 is known to be highly associated with an increased risk for T2D from multiple large population studies [19]. Here, we detected *TCF7L2* rs7903146 as one of the loci with the most highly increased H3K9ac enrichment in T2D (Table 2 and Figure 3D). Surprisingly, several *HLA* genes were also identified among the most altered H3K9ac enrichments in T2D, including *HLA-A, HLA-B, HLA-C, HLA-DRB1, HLA-DRB5, HLA-DQA1* and *HLA-DQB1* (Table S1 and Figure 3).

### 3.4. Regions of H3K9ac Changes Are Enriched for T2D Single-Nucleotide Polymorphisms (SNPs)

Genome-wide association studies (GWAS) in large population studies have led to the discovery of T2D-associated genetic variants loci, e.g., *PPARG* and *TCF7L2* [20,21]. The identified T2D SNPs are often located in non-coding regions and may colocalize with enhancers and promoters that are subject to epigenetic regulations. Since H3K9ac has been shown to be one of the major enhancer-associated chromatin modifications [22], we aimed to explore the overlap between the H3K9ac enrichment changes that we identified in T2D and the T2D SNPs that emerged from GWAS. To study this, we retrieved the GWAS summary statistics from the DIAGRAM [23]. We applied INterval enRICHment analysis (INRICH), an interval-based GWAS analysis tool, to map SNPs with their overlap regions of H3K9ac changes. Notably, we found significant associations between the T2D-specific H3K9ac enrichment that we identified and the T2D and T1D SNPs (Table 4). The most significant SNP loci are at rs7903146 in *TCF7L2* (*p* = 2.45E-39); and *HLA* SNPs including *HLA-B* (*p* = 0.0004)*, HLA-DQB1* (*p* = 0.0006)*, HLA-DRB1* (*p* = 0.001)*, HLA-DRB5* (*p* = 0.01) and *HLA-DQA1* (*p* = 0.02) (Table 4). These data underscore the significant association of H3K9ac changes in T2D with the GWAS SNPs. This relationship reinforced the biological relevance of epigenetic changes to the genetic factors impacting T2D.

### 3.5. Functional Pathways Related to T2D-Specific H3K9ac Enrichment Changes

We next examined the functional pathways related to T2D-specific H3K9ac enrichment changes. Categories of genes showing significantly increased or decreased H3K9ac (*p* < 0.05, Welch’s t test) in T2D included pathways related to a response to allograft rejection, cell adhesion molecules, ErbB signaling, T1D, autoimmune thyroid disease, graft-versus-host disease, endocytosis, antigen processing and presentation, Wnt signaling pathway, etc., the majority of which are linked to immune responses (Table 5 and Table S3). It has been suggested that hyperglycemia and oxidative stress in diabetes may accelerate the development of atherosclerosis; one mechanism for this could be via the promotion of immune reactions [24]. Our results on epigenetic changes in the immune response pathways in atherosclerotic patients with T2D may further support this view.

## 4. Discussion

Our study presents the first genome-wide profile of histone acetylation in humans affected with atherosclerosis and T2D. We performed ChIP-seq on PBMCs and traced the natural changes in how the T2D condition affects the epigenetic profile of atherosclerosis.

Previous studies have elucidated DNA methylation alterations in atherosclerosis [25], such as in vascular smooth muscle cells [26], aorta and coronary arteries [27], and aortic endothelial cells [28], as well as non-coding RNAs as epigenetic regulators [29]. The role of histone modification in atherosclerosis is, however, unclear. Several studies on cell lines and animal models have linked histone modifications to proinflammatory gene expression in atherosclerosis (detailed review by Khyzha et al. [30]). In human atherosclerotic plaques, changes in histone modifications were only supported by immunohistochemistry findings [31,32]. In this study, we investigated changes in histone modifications in PBMCs in association with atherosclerosis and T2D. PBMCs are clinically relevant cells with well-established roles in different diseases and previous studies have shown that PBMCs may provide disease-specific epigenetic signatures [33,34,35,36]. Therefore, an understanding of epigenetic changes linked to diabetes and atherosclerosis of PBMCs may contribute to identifying biologically promising epigenetic markers for pathogenesis of the diseases. To the best of our knowledge, no previous study has been performed to investigate genome-wide histone modification changes in atherosclerosis in humans, especially under diabetic conditions.

In this study, we investigated the modification of H3K9ac due to its association with T2D from previous studies. In T2D, despite the discovery of a large number of genetic loci associated with T2D by GWAS, the identified variants only explain a small proportion (~10%) of the heritability of T2D [37]. A growing body of evidence suggests that epigenetic mechanisms may contribute to explain the “missing heritability” in T2D. Epigenetic regulation via histone acetylation plays an important role in gene expression regulation and H3K9ac is commonly linked with gene activation by allowing genomic regions access to transcription machinery. In this study, we used ChIP-seq to map H3K9ac enrichment in PBMCs in T2D and ND patients with advanced cerebrovascular atherosclerosis and identified modifications at genomic regions unique to T2D. Notably, our analysis linked these epigenetic changes in T2D with genetics and pathways related to immunity. It is worth highlighting that we identified a genomic locus in *TCF7L2* at rs7903146 as one of the major sites for H3K9ac enrichment modifications in T2D. This specific locus in *TCF7L2* has been shown in multiple large population studies to be strongly associated with T2D risk [21]. Our novel finding, therefore, sheds light on the direct interaction between genetic and epigenetic mechanisms in T2D susceptibility. This is in line with previous studies where *TCF7L2* rs7903146 was identified to be significantly associated with angiographically diagnosed coronary artery disease (CAD) in the presence of T2D [38], and in PBMCs from CAD patients, where *TCF7L2* was identified as a key gene to be differentially expressed in CAD patients with T2D [39]. At this stage, we do not know if the H3K9ac enrichment pattern differs from risk- and non-risk-allele carriers at this specific locus, which requires further investigations.

Another surprising finding from our study is that we also identified several loci in the *HLA*s with most modifications in H3K9ac enrichment in T2D. Furthermore, we also found a significant association between T2D H3K9ac enrichment and pathways related to immunity, notably, T1D as one of the major identified pathways. The *HLAs* are reported to account for up to 50% of the familial aggregation of T1D, with the major genetic determinants in polymorphisms of class II *HLA* DQ and DR [40]. In our study, we identified several genetic loci in *HLA* that have been associated with T1D to be the most differentially enriched in H3K9ac, e.g., *HLA-B*, *HLA-DQB1*, *HLA-DRB*, *HLA-DRB5*, and *HLA-DQA1*. It has previously been shown that there is no fundamental difference between the immune activation and inflammation present in atherosclerosis among non-diabetics as compared to diabetics [24]. Albeit speculation, we may suggest that immune responses in T1D may also be operating in T2D, or that the two disease conditions may share common pathways.

Comparison of the ND vs. T2D ChIP-seq profile revealed a redistribution of H3K9ac with T2D. Previous in vitro models mimicking hyperinsulinemia showed that high insulin leads to a global increase in chromatin-associated histone acetylation, in particular at H3K9 [41]. An earlier investigation on PBMCs in T1D also demonstrated association between HbA1c level and H3K9ac enrichment [42]. In our study, all patients with T2D were diagnosed based on glycemic levels. We may only speculate at this stage that the epigenetic profile that we identified associated with T2D may be affected by both glucose and/or insulin levels; this requires further investigations.

In conclusion, our study provides fine mapping of genome-wide histone acetylation changes in patients with T2D and advanced atherosclerotic disease. By identifying loci linked to T2D and T1D genetics, we revealed the potential mechanisms of epigenetic and genetic interactions. Furthermore, we also suggest epigenetic modifications in pathways related to immunity in T2D. These findings open the possibility that prevention of T2D-dysregulation at the chromatin level may present novel therapeutic avenues for T2D.

## Figures and Tables

**Table 1:** Categorical variables are expressed in total amount and percentages. Continuous variables as median and interquartile range (IQR) or mean and standard deviation (SD). * BMI, body mass index. ^†^ hsCRP, high-sensitivity CRP. ^‡^ LDL, low-density lipoprotein. ^§^ HDL, high-density lipoprotein. ^||^ HbA1c, hemoglobin A1c, was available for 67% (*n* = 12) of the cohort. Hypertension defined as: anti-hypertensive treatment or systolic pressure > 140 mmHg. Level of significance between no diabetes and T2D patients is marked by *** *p* < 0.005.

	All	No T2D (*n* = 10)	T2D (*n* = 8)
Age, years (IQR)	69.5 (61.8–77.3)	69.5 (62.8–73.3)	69.5 (59.5–78.8)
Sex, males (%)	12 (67)	6 (60)	6 (75)
Smoking, current and previous/non smokers (%)	16/2 (89/11)	9/1 (90/10)	7/1 (88/12)
BMI (IQR) *	26.8 (24.1–28.8)	26.4 (24.1–30.0)	27.0 (24.6–27.7)
C-peptide (pmol/L)	1558.5 (253.6–3666.2)	1471.0 (464.4–3588.4)	1668.0 (253.6–3666.2)
Degree of stenosis, % (IQR)	87.5 (78.8–90.0)	90 (83.4–91.3)	85 (71.3–90.0)
Hypertension, *n*(%)	14 (78)	9 (90)	5 (63)
Blood markers			
hsCRP, mg/L (IQR) ^†^	3.7 (2.7–5.0)	3.7 (2.5–4.7)	3.6 (2.2–6.0)
HbA1c, mmol/mol (IQR) ^||^	49.5 (46.3–74.2)	45 (38–47)	64 (49–77) ***
Total cholesterol, mmol/L (IQR)	4.4 (3.7–5.4)	4.6 (3.8–5.0)	4.2 (3.4–6.0)
LDL, mmol/L (IQR) ^‡^	2.6 (1.8–3.4)	2.2 (1.8–2.9)	2.7 (1.9–4.0)
HDL, mmol/L (IQR) ^§^	1.0 (0.8–1.3)	1.1 (0.8–1.8)	1.0 (0.8–1.1)
Triglycerides, mmol/L (IQR)	1.7 (1.0–2.2)	1.6 (0.9–2.2)	1.7 (1.0–2.9)
Blood pressure lowering treatment, *n*(%)			
RAS inhibitor	10 (56)	6 (60)	4 (50)
Beta blocker	10 (56)	7 (70)	3 (38)
Blood glucose lowering treatment, *n*(%)			
Lifestyle changes	1 (6)	-	1 (13)
Oral glucose lowering treatment	4 (22)	-	4 (50)
Insulin only	0 (0)	-	0 (0)
Insulin and oral glucose lowering	2 (11)	-	2 (25)
Statin treatment, *n*(%)	18 (100)	10 (100)	8 (100)

**Table 2:** Top genetic loci with increased H3K9ac enrichment in atherosclerosis patients with T2D.

Gene Symbol	Peak Start	Peak End	M_Value_Rescaled	A_Value_Rescaled	−log_10_ (*p*-Value)
*LOC100631378*	38327931	38328189	−5.49	2.11	7.14
*HCG4B*	1188949	1189493	−3.34	3.92	8.81
*CHRM2*	136684799	136685143	−2.74	4.82	11.19
*LOC349160*	136684799	136685143	−2.74	4.82	11.19
*TCF7L2*	114757868	114758883	−2.66	18.27	Inf
*HLA-C*	2586205	2587065	−2.33	4.88	8.52
*CATSPERB*	92107659	92108000	−2.29	3.56	3.46
*CECR2*	18024490	18024867	−2.25	5.16	9.75
*XKR6*	10790323	10790692	−2.17	4.20	4.48
*DPP6*	154129998	154130240	−2.15	4.10	4.27

**Table 3:** Top genetic loci with decreased H3K9ac enrichment in atherosclerosis patients with T2D.

Gene Symbol	Peak Start	Peak End	M_Value_Rescaled	A_Value_Rescaled	−log_10_ (*p*-Value)
*GPSM3*	3630151	3630907	5.33	2.66	Inf
*RNF5P1*	3616969	3617540	5.17	2.58	Inf
*RNF5*	3616969	3617540	5.17	2.58	Inf
*APOM*	3129514	3130353	4.78	2.39	Inf
*BAG6*	3129514	3130353	4.78	2.39	Inf
*HCP5*	2940084	2941136	4.21	2.10	5.72
*SERF1B*	70195996	70198315	4.03	2.02	5.18
*SERF1A*	70195996	70198315	4.03	2.02	5.18
*ATP6V1G2-DDX39B*	2888815	2890051	3.83	3.50	10.29
*DDX39B*	2888815	2890051	3.83	3.50	10.29

**Table 4:** T2D-associated genetic variant loci with overlap regions of H3K9ac changes.

GENE	CHR	START	STOP	ZSTAT	*p-*Value
*TCF7L2*	10	114757867	114758883	13.07	2.45E-39
*TCF7L2*	10	114757868	114758883	13.07	2.45E-39
*HLA-B*	6	31322760	31325963	3.33	0.00044
*HLA-DQB1*	6	32631728	32636147	3.19	0.00070
*HLA-B*	6	31320376	31326175	3.09	0.0010
*HLA-DRB1*	6	32551226	32558284	3.04	0.0012
*HCG27*	6	31164981	31166290	2.96	0.0015
*HLA-DRB1*	6	32551453	32552934	2.89	0.0019
*HCG27*	6	31164908	31166411	2.87	0.0021
*XKR6*	8	10790323	10790692	2.33	0.0099
*HLA-DRB5*	6	32496119	32498185	2.23	0.011
*MAGI1*	3	65678931	65679421	1.95	0.026
*HLA-DQA1*	6	32604890	32606948	1.92	0.027
*PKD2L1*	10	102055647	102056160	1.88	0.030
*CA5A*	16	87933312	87933806	1.88	0.030
*HLA-DQA1*	6	32604815	32607091	1.85	0.032
*DEPTOR*	8	120994091	120994421	1.85	0.032

**Table 5:** Functional pathways related to T2D-specific H3K9ac enrichment changes.

Pathway	Peak Related Genes with Pathway Annotation	*p-*Value	Q-Value
Allograft rejection	4 (4%)	0.00032	0.025
Cell adhesion molecules (CAMs)	7 (7%)	0.00050	0.025
ErbB signaling pathway	6 (6%)	0.00053	0.025
Type I diabetes mellitus	4 (4%)	0.00058	0.025
Autoimmune thyroid disease	4 (4%)	0.0010	0.029
Graft-versus-host disease	4 (4%)	0.0010	0.029
Endocytosis	9 (9%)	0.0017	0.043
Asthma	3 (3%)	0.0019	0.043
Acute myeloid leukemia	4 (4%)	0.0034	0.067
Intestinal immune network for IgA production	3 (3%)	0.0052	0.092
Antigen processing and presentation	4 (4%)	0.0074	0.11
Herpes simplex infection	6 (6%)	0.0078	0.11
Viral myocarditis	4 (4%)	0.0087	0.18
Aldosterone synthesis and secretion	4 (4%)	0.0099	0.12
Epstein–Barr virus infection	6 (6%)	0.012	0.15
Inflammatory bowel disease (IBD)	3 (3%)	0.015	0.16
Wnt signaling pathway	5 (5%)	0.016	0.16
Influenza A	6 (6%)	0.016	0.16
Endometrial cancer	3 (3%)	0.017	0.16
EGFR tyrosine kinase inhibitor resistance	4 (4%)	0.018	0.16
Cholinergic synapse	4 (4%)	0.019	0.16
PI3K-Akt signaling pathway	8 (8%)	0.021	0.17
HTLV-I infection	6 (6%)	0.024	0.18
Leishmaniasis	3 (3%)	0.025	0.18
Rheumatoid arthritis	3 (3%)	0.030	0.21
Inositol phosphate metabolism	3 (3%)	0.033	0.23
Dopaminergic synapse	4 (4%)	0.037	0.24
Systemic lupus erythematosus	4 (4%)	0.038	0.24
Proteoglycans in cancer	7 (7%)	0.041	0.25
MicroRNAs in cancer	5 (5%)	0.045	0.26

## Data Availability

The data underlying this article will be shared on reasonable request and in compliance with the appropriate GDPR regulations.

## Supplementary Materials

The following are available online at https://www.mdpi.com/article/10.3390/biomedicines9121908/s1, Table S1: Increaed H3K9ac in T2D, Table S2: Decreaed H3K9ac in T2D, Table S3: Group2b-VS-group4.
